# eIF3 interacts with histone H4 messenger RNA to regulate its translation

**DOI:** 10.1016/j.jbc.2021.100578

**Published:** 2021-03-23

**Authors:** Hassan Hayek, Lauriane Gross, Aurélie Janvier, Laure Schaeffer, Franck Martin, Gilbert Eriani, Christine Allmang

**Affiliations:** Architecture et Réactivité de l’ARN, Centre National de la Recherche Scientifique, Institut de Biologie Moléculaire et Cellulaire, Université de Strasbourg, Strasbourg, France

**Keywords:** eukaryotic initiation factor, histone mRNA, translation regulation, translation initiation, protein synthesis, RNA–protein interaction, RNA structure, 4E-SE, eIF4E-sensitive element, 5’ UTR, 5’ untranslated region, CMCT, 1-cyclohexyl-3-(2-morpholinoethyl)carbodiimide metho-p-toluene sulfonate, DMEM, Dulbecco’s modified Eagle media, eIF, eukaryotic initiation factor, GST, glutathione-S-transferase, HLH, helix-loop-helix, IEF, isoelectric focusing, IRES, internal ribosomal entry site, RNP, ribonucleoprotein immunoprecipitation, RRM, RNA recognition motif, SHAPE, selective 2’-hydroxyl acylation analyzed by primer extension, SLBP, stem-loop binding protein, TWJ, three-way junction

## Abstract

In eukaryotes, various alternative translation initiation mechanisms have been unveiled for the translation of specific mRNAs. Some do not conform to the conventional scanning-initiation model. Translation initiation of histone H4 mRNA combines both canonical (cap-dependent) and viral initiation strategies (no-scanning, internal recruitment of initiation factors). Specific H4 mRNA structures tether the translation machinery directly onto the initiation codon and allow massive production of histone H4 during the S phase of the cell cycle. The human eukaryotic translation initiation factor 3 (eIF3), composed of 13 subunits (a-m), was shown to selectively recruit and control the expression of several cellular mRNAs. Whether eIF3 mediates H4 mRNA translation remains to be elucidated. Here, we report that eIF3 binds to a stem-loop structure (eIF3-BS) located in the coding region of H4 mRNA. Combining cross-linking and ribonucleoprotein immunoprecipitation experiments *in vivo* and *in vitro*, we also found that eIF3 binds to H1, H2A, H2B, and H3 histone mRNAs. We identified direct contacts between eIF3c, d, e, g subunits, and histone mRNAs but observed distinct interaction patterns to each histone mRNA. Our results show that eIF3 depletion *in vivo* reduces histone mRNA binding and modulates histone neosynthesis, suggesting that synthesis of histones is sensitive to the levels of eIF3. Thus, we provide evidence that eIF3 acts as a regulator of histone translation.

In eukaryotes, translation initiation requires multiple complexes of eukaryotic initiation factors (eIFs) to assemble elongation-competent ribosomes to the mRNA (1, 2). The recognition of the m^7^G cap structure by the eIF4E-binding factor that is part of the translation initiation complex eIF4F (composed of the three subunits eIF4E, eIF4A, and eIF4G) constitutes the first step of the canonical translation initiation and is a prerequisite to ribosomal attachment (3, 4, 5). The initiation codon is then recognized by a scanning mechanism of the mRNA by the initiator tRNAi^Met^ linked to the 40S subunit (43S complex). eIF3, the largest multisubunit initiation factor, has been implicated in events throughout the initiation pathway (6, 7, 8, 9, 10). Bound to the 40S subunit near both the mRNA entry and exit channels, it participates to the stabilization of the 43S preinitiation complex (PIC), to its recruitment to the mRNA (8, 11, 12) and interacts with the eIF4F complex. In recent years, a remarkable diversity in the recruitment of eukaryotic ribosomes by mRNAs has been unveiled (13, 14). This is the case for viruses that have developed simplified systems to improve translation efficiency, allowing also hijacking of the host translation machinery for their own mRNA. Namely, internal ribosomal entry sites (IRESs), located in the 5’ untranslated region (5’ UTR) of viral mRNA, enable to initiate translation with only a partial set of eIFs in a cap-independent manner sometimes even without any scanning step (1, 13, 15, 16).

Translation initiation of histone H4 mRNA is an alternative initiation mechanism combining canonical (cap dependence) and IRES-like initiation strategies (no-scanning, internal recruitment of initiation factors) (17, 18). H4 mRNA contains specific RNA structures that tether the translation machinery directly on the AUG initiation codon. A double stem-loop structure called eIF4E-sensitive element (4E-SE) binds eIF4E without the need of the cap, and a three-way junction (TWJ) sequesters the m^7^G cap and facilitates direct 80S ribosomes positioning to the cognate AUG start codon (17). The lack of scanning appears to promote high expression levels of histone H4 protein during the S-phase of the cell cycle for rapid incorporation into nucleosomes. The cryo-EM structure of 80S ribosome in complex with H4 mRNA showed that the TWJ forms a repressive structure at the mRNA entry site on the 40S subunit next to the tip of helix 16 of 18S ribosomal RNA (rRNA) (18). H4 mRNA harbors a sequence complementary with the h16 loop of the 18S rRNA, which tethers the mRNA to the ribosome to promote proper start codon positioning (18). This highlights the functional importance of the H4 mRNA structures located in the coding sequence during the initiation process. An additional secondary H4 mRNA structure, also located in the coding sequence, was recently found to interact with eIF3 (19).

The initiation factor eIF3 is capable of selectively recruiting and controlling the expression of several cellular mRNAs by binding to specific stem loops (19, 20, 21). This regulation occurs primarily through interactions with 5’UTR structural elements, but the role of eIF3 in regulation is not yet clearly established (19, 22), nor is the mechanism by which eIF3 selects its mRNA targets. Composed of 13 subunits (a-m), the structural scaffold of mammalian eIF3 is a multilobed octamer conserved in the proteasome and signalosome complexes (11, 23, 24). Six eIF3 subunits (a, c, e, k, l, and m) bear PCI (**P**roteasome, **C**OP9, e**I**F3) and two subunits (f, h) bear MPN (**M**pr1–**P**ad1 **N**-terminal) signature domains. eIF3d seems to be located in a peripheral position, is not required for the integrity of the complex and not conserved across species but is essential in some organisms (23). Near-atomic resolution structure of the human eIF3 in the context of the 48S recently revealed that eIF3d interacts both with the 40S and the octameric core, as well as potentially with eIF3F (24). eIF3d was also shown to bind the 5’ cap of some specific mRNAs in a way reminiscent of eIF4E suggesting the existence of a second mechanism of cap-dependent translation, linked to eIF3d (20, 25). Peripheral subunits of eIF3 also include the eIF3b, g, i module that encircles the 40S and connects the mRNA entry channel to the exit site of the ribosome (24, 26, 27). Due to the presence of several RNA-binding domains, eIF3 offers multiple opportunities of interactions with its targets.

Here we report that eIF3 binds to a stem-loop structure located in the coding region of H4 mRNA downstream of the 4E-SE. Combining cross-linking and ribonucleoprotein immunoprecipitation (RNP IP) *in vivo* and *in vitro*, we found that eIF3 interacts with H4 and also with H1, H2A, H2B, and H3 histone mRNAs. We demonstrate a direct interaction of H4 mRNA with eIF3c, d, e and g subunits and suggest the existence of different interaction patterns for the different histone mRNAs. After having inactivated eIF3 *in vivo* by siRNA interference in G1/S synchronized cells, we selectively monitored histone neosynthesis by [^35^S] pulse labeling. These experiments reveal that eIF3 could act as a modulator of histones translation particularly in metabolic conditions where eIF3 comes to be limiting.

## Results

### eIF3 interacts with a stem-loop structure in the coding sequence of H4 mRNA

**Figure 1:**
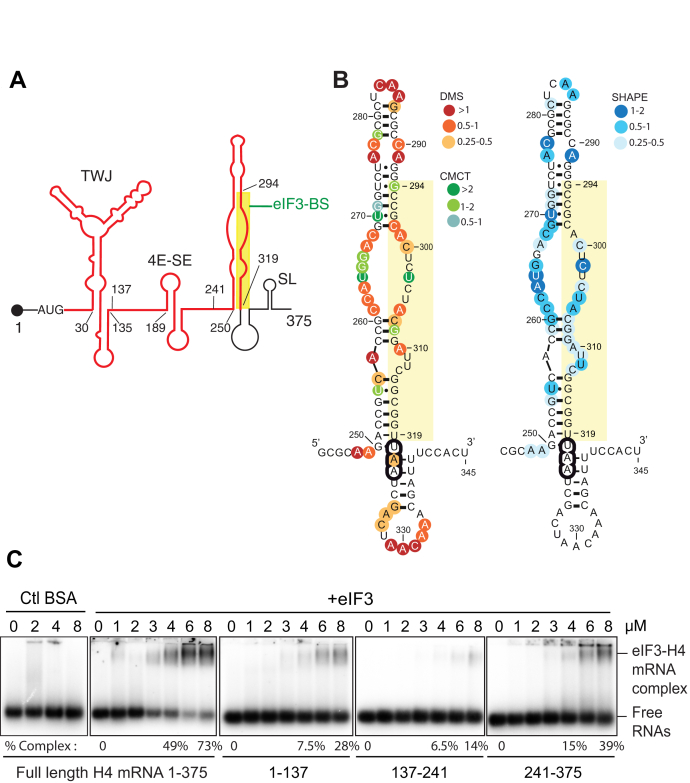
**The eIF3 complex interacts with a stem-loop structure in the coding region of H4 mRNA.***A*, secondary structure model of the 375 nucleotides mouse histone H4 mRNA (17, 77). H4 mRNA is characterized by three functional structural elements. The TWJ (Three-Way Junction) is the cap-binding site and allows ribosome positioning (nts 30–135) while the eIF4E-sensitive element (4E-SE) structure (189–249) recruits eIF4E. The region of 25 nucleotides interacting with eIF3 and identified by PAR-CLIP experiments (nts 294–319) is boxed in *yellow* (19) and part of a stem-loop structure (nts 250–320) called eIF3-binding site (eIF3-BS). The 5’UTR and 3’ UTR are represented in *black* and the coding region in *red*, the AUG codon is indicated; the 3’UTR contains a highly conserved 16 nt stem-loop structure (SL) that interacts with the stem-loop binding protein (SLBP) for processing, export and translational of the mRNA. *B*, secondary structure of nucleotides 244 to 345 summarizing the H4 mRNA solution structure probing results obtained by chemical probing (DMS and CMCT) and selective 2’-hydroxyl acylation analyzed by primer extension (SHAPE). Reactivities are shown as averages from three independent experiments (except for CMCT: average from two experiments). SHAPE reactivities could not be determined for nts 313 to 345. *C*, electromobility shift assays (EMSA) analysis of eIF3 binding to H4 mRNA. [α^32^P]-GTP internally radiolabeled full-length H4 mRNA (1–375) or truncated H4 mRNA transcripts (1–137, 137–241 and 241–375) were incubated in the presence of increasing amounts of purified eIF3 complex (1–8 μM). eIF3 was omitted in the 0 μM lanes. BSA (2, 4, and 8 μM) was used as a control (lanes 2–4). The complexes were separated on 0.7% agarose gel under native conditions. The percentage of H4 mRNA in complex with eIF3 is indicated below the gels for 4 and 8 μM of eIF3.

It was previously established that human translation initiation factor eIF3 can target mRNAs in a transcript-specific manner and can function as an activator or repressor of translation (19, 20, 21). The majority of the mRNAs identified contain a single eIF3-binding site predominantly located within 5’UTR RNA structural elements (19). By PAR-CLIP a 25 nt H4 mRNA sequence was identified among eIF3 mRNA targets interacting with eIF3 (19). By contrast, this sequence is located in the coding region of H4 mRNA between nucleotides 294 and 319 (Fig. 1*A*) in a region adjacent to previously characterized structural elements, namely the TWJ and the 4E-SE (17). We determined the secondary structure of H4 mRNA around the potential eIF3-binding site using chemical probing and selective 2’-hydroxyl acylation analyzed by primer extension (SHAPE) (Fig. 1*B*). Chemical probing and SHAPE revealed that the potential eIF3-binding site maps to the 3’ strand of a 70 nt long stem-loop structure named hereafter eIF3-binding site (eIF3-BS). The primer extension pattern revealed the presence of a large central bulge encompassing nts (261–269 and 299–306) in addition to the two small bulges (ΔG = −28.6 kcal/mol at 37 °C, from nts 250 to 320 (28)). Fourteen nts of the sequence identified by PAR-CLIP (19) were found in the double-stranded part of the motif (Fig. 1*B*) in agreement with an interaction of eIF3 occurring in the context of an RNA secondary structure. To evaluate the importance of eIF3-BS, RNA-electrophoretic mobility shift assays were performed using purified full-length H4 mRNA (H4 FL) and three truncated radiolabeled H4 RNA fragments (H4 1–137, H4 137–241, and H4 241–375) generated by *in vitro* transcription. H4 1 to 137 contains the TWJ, H4 137 to 241 the 4E-SE, and H4 241 to 375 contains the eIF3-BS. Prior to complex formation RNAs were heat denaturated and refolded to promote formation of secondary and tertiary structures. Purified eIF3 complex directly interacted with H4 FL with an estimated Kd of 4 μM. eIF3 moderately interacted with H4 241 to 375 (eIF3-BS) but also with H4 1 to 137 (TWJ) and shifted 39% and 28% of the RNAs respectively at high concentrations of eIF3 (Fig. 1*C*). In the same conditions only a weak 14% band shift was observed for H4 137 to 241 (Fig. 1*C*). No retarded complex was obtained in the presence of BSA, used as a negative control. The major eIF3-binding site therefore seems to reside in the eIF3-BS fragment but weaker eIF3 binding can also occur in the H4 1 to 137 fragment, which includes the TWJ. Optimal eIF3 binding therefore seems to require the full-length mRNA. Altogether these results confirm that eIF3 interacts *in vitro* with the histone H4 mRNA and that the PAR-CLIP defined sequence belongs to the eIF3-BS stem-loop structure.

### eIF3 interacts *in vivo* with histone mRNAs

**Figure 2:**
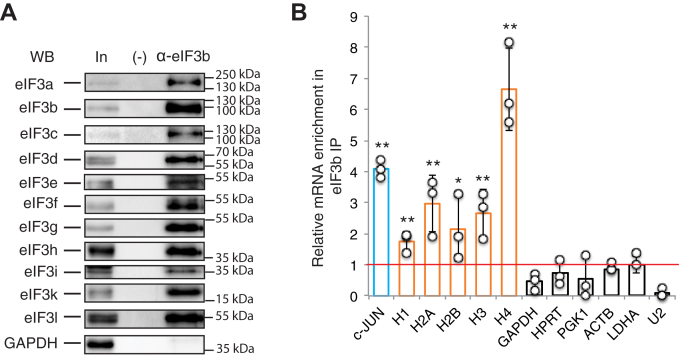
**The eIF3 complex interacts with histone mRNAs *in vivo*.** Immunoprecipitation of the endogenous eIF3 complex and its associated RNAs from HEK293FT cells using antibodies against eIF3b (α-eIF3b). *A*, analysis of the endogenous immunoprecipitated proteins by SDS-PAGE and western blotting using antibodies against the indicated proteins. In: Input (5% of total); (-): Control without antibodies. The position of the molecular weight markers is indicated. *B*, analysis of mRNAs interacting with eIF3. The relative mRNA enrichment in the eIF3b IP was measured by qRT-PCR and determined by the *ΔΔCt* method using LDHA mRNA as a normalizer. *Circles* represent values obtained in three independent experiments and the *bars* correspond to the mean. c-JUN mRNA (*blue bar*) is a previously characterized eIF3 target (19). H1, H2A, H2B, H3, and H4 (*orange bars*) are histone mRNAs. GAPDH, HPRT, PGK1, ACTB, and LDHA are canonical mRNAs and snRNA U2 is a negative control (*white bars*). Error bars represent the standard deviation of the three independent experiments. The *horizontal line* represents the level of LDHA mRNA binding (5% in average, normalized to 1). *Asterisks* indicate statistically significant differences with the LDHA control mRNA. ∗*p* < 0.05 and ∗∗*p* < 0.005 based on Student’s *t*-test.

To determine if eIF3 is capable of interacting with all histone mRNAs, we immunoprecipitated eIF3-RNA complexes from HEK293FT cells. Formaldehyde cross-linking was used to stabilize transient interactions and minimize RNP complexes rearrangements (29). The full endogenous eIF3 complex thus stabilized was immunoprecipitated using an antibody directed against the eIF3b subunit (19, 30). Western blotting revealed that 11 of the 13 eIF3 subunits (a, b, c, d, e, f, g, h, i, k, l) were specifically co-immunoprecipitated (Fig. 2*A*). No interaction was detected for GAPDH used as a negative control. The RNAs associated with eIF3 were determined by qRT-PCR (Fig. 2*B*). The c-JUN mRNA is a target of eIF3 in PAR-CLIP experiments and was used as positive control (19). The housekeeping, nonhistone mRNAs GAPDH, HPRT, PGK1, ACTB, and LDHA undergo canonical cap-dependent translation (20). The spliceosomal U2 snRNA was used as a negative control. On average 5% of the housekeeping mRNAs were retained in the anti-eIF3b immunoprecipitation, whereas only 0.2% of U2 snRNA was detected, reflecting the general role of eIF3 in mRNA translation. The relative mRNA enrichment in the eIF3b IP was normalized against that obtained for LDHA mRNA. As expected c-JUN mRNA was co-immunoprecipitated by eIF3 and enriched 4.5 times in the IP compared with LDHA, whereas this is not the case for GAPDH, HPRT, PGK1, and ACTB control mRNAs; this is in accordance with previous results (19). Our results indicate that all histone mRNAs are significantly enriched in the anti-eIF3b immunoprecipitation. The binding of histone H4 mRNA is the highest with sixfold while histones H1, H2A, H2B, and H3 are enriched between 1.7- and 3-fold (Fig. 2*B*). On average these results are similar to those observed for c-JUN and confirm that histone mRNAs are prime targets of eIF3.

### Identification of eIF3 subunits that interact with H4 mRNA by *in vitro* cross-linking

**Figure 3:**
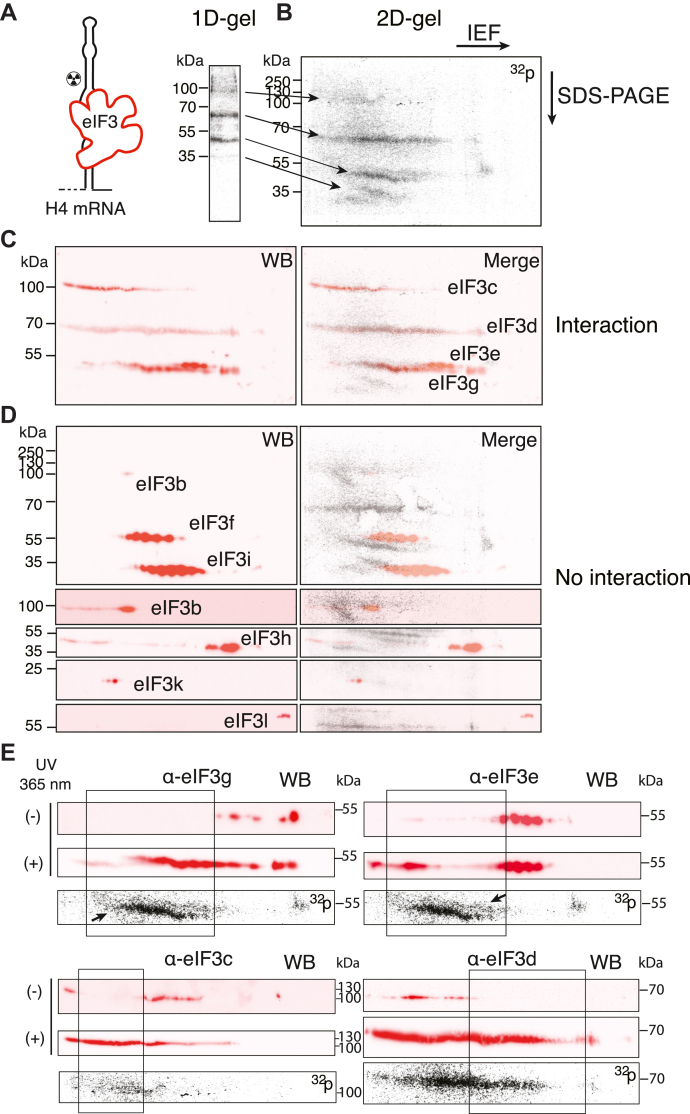
**Identification of eIF3 subunits in interaction with H4 mRNA by UV cross-linking and western blotting.***A*, schematic representation of UV cross-linking of [α^32^P]-ATP radiolabeled thioU-H4 mRNA in the presence of purified eIF3 complex at 365 nm. After RNase A digestion, radioactive mRNA fragments protected against degradation remain cross-linked to eIF3 subunits. [^32^P]-labeled proteins are resolved by SDS-PAGE (*A*) or 2D-gel electrophoresis (*B*). In the first dimension proteins were separated by isoelectric focusing (IEF) pH 4 to 7 followed by SDS-PAGE in the second dimension. Radiolabeled proteins are transferred to PVDF membranes, revealed by Phosphorimaging and subjected to western blot analysis (WB) using antibodies directed against individual eIF3 subunits. *C* and *D*, identification of cross-linked proteins by superimposition of WB signals (*red signals*, *left panels*) and radioactive signals (Merge, *right panels*). Six replicate experiments have been performed and individual antibodies have been probed in triplicates. For clarity all the positive interaction signals have been compiled on the PVDF membrane represented in panel *C* while negative interaction signals have been compiled on a different membrane in panel *D*, to this end antibodies were used successively. *C*, the superimposition of WB and radioactivity signals indicates that the eIF3 subunits c, d, e, and g interact with the H4 mRNA. The PVDF membrane in panel *B* was used for the WB probing shown in panel *C*. *D*, no superimposition and therefore no interaction were detected between the eIF3 subunits b, f, h, i, k, and l and H4 mRNA. *E*, comparison of the migration profiles of eIF3g, e, c, and d proteins on 2D gel in the presence and absence of H4 mRNA crosslinking. WB (*red signals*, *upper panels*) of control experiments realized without cross-linking (-) are compared with cross-linking conditions (+). The cross-link of eIF3 subunits to H4 mRNA induces a shift of the WB signal to the *boxed area*. Radiolabeled proteins are revealed by Phosphorimaging (*lower gray*^*32*^*P panels*). The first ^32^P panels are duplicate images. They correspond to the same PVDF membrane that was probed with α-eIF3g and α-eIF3e antibodies. *Arrows* point to the position of the radioactive cross-linking signal corresponding to eIF3g and e. The position of the molecular weight markers is indicated.

Different modes of interaction between the eIF3 complex and its RNA targets have been established. RNA-binding domains have been identified in the eIF3a, b, and g subunits (31) while eIF3d is capable to bind the cap of several mRNAs by a dedicated cap-binding domain (20). Only the eIF3a, b, and c subunits bind IRES elements (1, 32, 33) while eIF3 mRNA targets identified by PAR-CLIP interact with distinct combinations of the eIF3a, b, d, and g subunits (19). In order to precisely identify the subunits of the eIF3 complex in direct interaction with the H4 mRNA, we performed UV cross-linking experiments using a uniformly radiolabeled ThioU-H4 mRNA transcript in the presence of purified eIF3 complex (Fig. 3). After RNase A digestion, only radioactive mRNA fragments protected against degradation because of their interaction with eIF3 remained cross-linked to eIF3 subunits. Separation of the cross-linked products by denaturing gel electrophoresis revealed radiolabeling of at least four different eIF3 subunits with apparent molecular weights of 110, 65, 50, and 45 kDa (Fig. 3*A*). Several eIF3 subunits share similar molecular weight. This is the case for the subunits eIF3a, b, and c (110 kDa), eIF3d and l (65 kDa) as well as eIF3e, f, and g (45 kDa). To identify the radiolabeled eIF3 subunits, cross-linked products were separated by two-dimensional gel electrophoresis (2D-gel) (Fig. 3*B*) followed by western blot analysis using antibodies directed against 11 of the 13 eIF3 subunits (Fig. 3, *C* and *D*). Our results show cross-linking signals between the subunits eIF3c, d, e, and g (Fig. 3*C*) and H4 mRNA for which radioactivity and western blot signals overlay. eIF3 subunits undergo numerous posttranslational modifications (34), this is reflected by the dotted migration profile on the 2D-gel, each dot corresponding to the different levels of modifications, and thus isoelectric charge of the protein (Fig. 3, *C*–*E*). The migration profile of the proteins is partially shifted upon cross-linking and spreads over a wide range of pH due to the presence of the additional charges coming from the cross-linked RNA moiety (Fig. 3*E*). By contrast, the western blot signals of the eIF3 subunits b, f, h, i, k do not overlap with the radioactivity signals, showing that they do not interact with H4 mRNA (Fig. 3*D*).

### eIF3c, d, e, and g subunits interact with histone mRNAs *in vitro*

**Figure 4:**
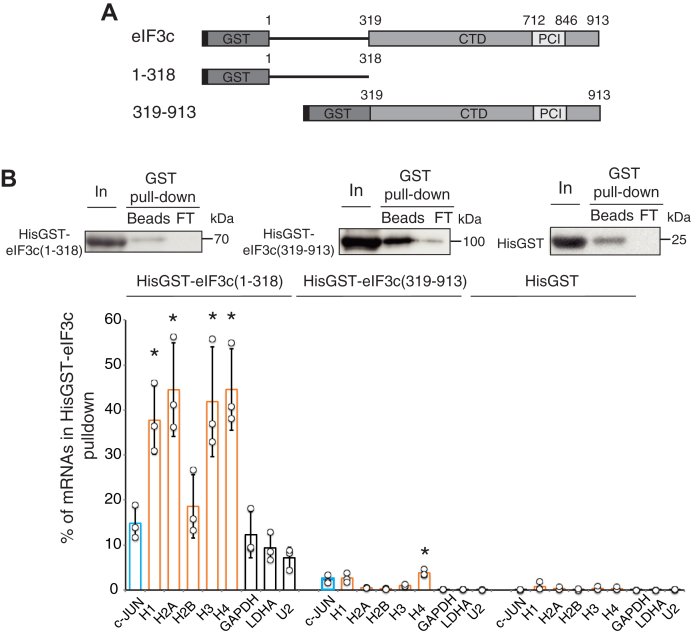
**The N-terminal domain of eIF3c interacts with histone mRNAs.***A*, schematic representation of eIF3c (1–913) and truncated recombinant eIF3c fused to hexa-histidine and glutathione S-transferase (HisGST). eIF3c contains a PCI (Proteasome, COP9/signalosome, eIF3) motif (712–846) in the C-terminal region (CTD). eIF3c (1–318) covers the N-terminal region and eIF3c (319–913) corresponds to the CTD. *B*, GST pull-down experiments were performed using the HisGST proteins and total RNA extracted from HEK293FT cells. Western blot panels confirm binding of the HisGST target proteins to GST-trap matrix (Beads). In: Input (5% of total); Beads: 10% of bound protein; FT: 5% effluent. The position of the molecular weight markers is indicated to the *right of the panels*. Bound RNAs were analyzed by qRT-PCR and are as described in Figure 2*B*. The histograms represent the % of mRNAs in GST pull-down compared with the input. *Circles* represent values obtained in independent experiments and *bars* correspond to the mean. *Blue bars*, c-JUN mRNA; *orange bars*, histone mRNAs; *white bars*, control GAPDH, LDHA mRNAs and U2 snRNA. The error bars represent the standard deviation of three biological replicates. *Asterisks* indicate statistically significant differences with the LDHA control mRNA. ∗*p* < 0.05 and ∗∗*p* < 0.005 based on Student’s *t*-test.

**Figure 5:**
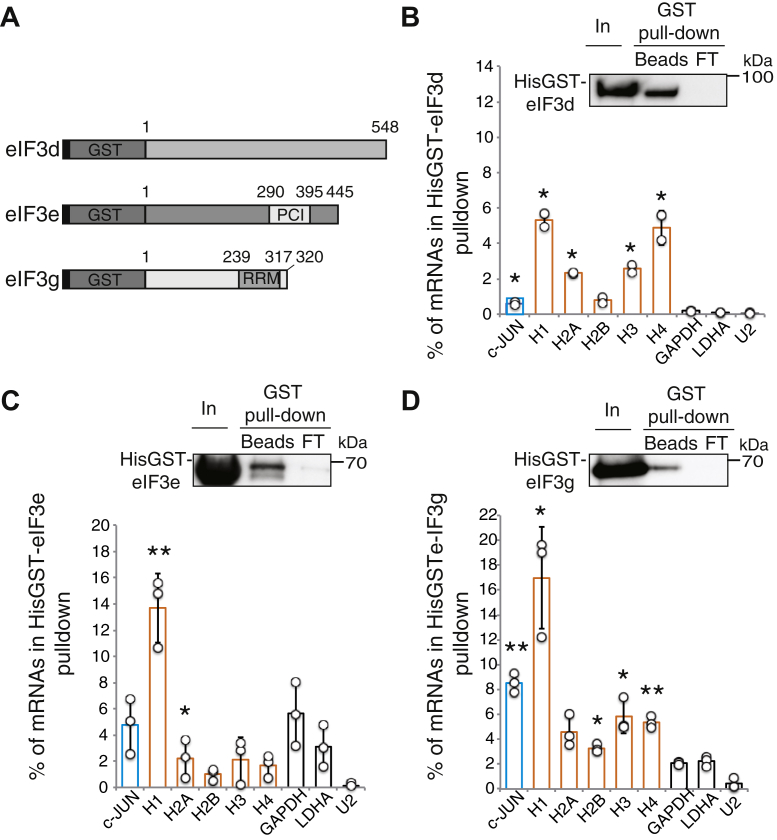
**HisGSTeIF3 d, e, and g interact with histone mRNAs.***A*, schematic representation of HisGST-eIF3d, HisGST-eIF3e, and HisGST-eIF3g. eIF3e contains a C-terminal PCI domain and eIF3g a C-terminal RNA Recognition Motif (RRM). *B*–*D*, GST pull-down results for HisGST-eIF3d, HisGST-eIF3e, and HisGST-eIF3g. Data are presented as described in Figure 4.

The four subunits eIF3c, d, e, and g that we have identified in interaction with H4 mRNA play important roles in the formation and positioning of the eIF3 complex in the 80S ribosome. eIF3c and e subunits belong to the structural core of eIF3. Only eIF3g has an RNA Recognition Motif (RRM) (35) and eIF3d binds to the cap of certain mRNAs (20, 25). Both eIF3g and d are part of the peripheral module of eIF3 and are flexibly linked to the structural core, mainly through interactions between the eIF3d and eIF3e subunit (12, 36, 37). We performed glutathione-S-transferase (GST) pull-down experiments using total RNA from HEK293FT cells and recombinant HisGST eIF3c, d, e, and g proteins to test their ability to interact directly and independently of the eIF3 complex with histone mRNAs. Unlike HisGST-eIF3d, e, and g proteins, soluble full-length recombinant HisGST-eIF3c could not be produced (32). We therefore produced N-terminal and C-terminal truncated HisGST-eIF3c 1 to 318 and HisGST-eIF3c 319 to 913 (Fig. 4*A*). The binding of histone mRNAs (H1, H2A, H2B, H3, and H4) with the recombinant HisGSTeIF3 proteins was analyzed by qRT-PCR (Fig. 4*B*). As previously described, the eIF3 target c-JUN mRNA was used as a positive control, while the housekeeping mRNAs (GAPDH, LDHA) and snRNA U2 were used as negative controls. On average, less than 1% of the mRNA tested interacted with a HisGST control protein, these values were subtracted from the data. The N-terminal fragment HisGST-eIF3c 1 to 318 preferentially interacted with the mRNAs of histones H1, H2A, H3, and H4 for which 38 to 44% of the mRNAs were recovered in the bound fraction. These values are approximately 3 to 4 times higher than that observed for c-JUN mRNA (15%) or housekeeping mRNAs (10%). Levels of binding to histone H2B mRNA (18%) were comparable to those of the positive control mRNA. In sharp contrast, the C-terminal region of eIF3c does not interact with the mRNAs tested and only 3% of the histone mRNAs were pulled-down by HisGST-eIF3c 319 to 913. These results suggest that the mRNA-binding domain of eIF3c is located in the N-terminal region of eIF3c 1 to 318 and interacts preferentially with histone mRNAs. The conserved N-terminus of eIF3c also contributes to eIF5 and eIF1 binding. Recent structural data showed that human eIF1 interacts with the conserved mammalian-specific residues 166 to 287 (24), thus revealing a dual RNA and protein-binding activity for the N-terminal region of eIF3c. Strikingly GST pull-down experiments revealed differential interaction patterns for the individual eIF3 subunits tested. HisGSTeIF3d interacts with all the histone mRNAs tested. Between 3 and 5% of H1, H2A, H3, and H4 mRNAs but only 1% of H2B mRNAs and c-JUN mRNAs are bound by the protein (Fig. 5*B*). Although modest, these binding levels are 10 to 50 times higher than those observed for the control mRNAs GAPDH, LDHA, and snRNA U2, which are 0.1% on average. In contrast, HisGSTeIF3e interacts only with histone H1 mRNA (14% of bound mRNA) but not with H2A, H2B, H3, and H4 mRNAs. For the latter the interaction rates are even lower than that observed for the housekeeping control mRNAs and c-JUN mRNA (Fig. 5*C*). Finally, HisGSTeIF3g reveals an intermediate profile and binds to all the histone mRNAs tested, with a strong preference for the H1 mRNA for which 17% of the mRNA is retained. HisGSTeIF3g also retains 5% of the other histone mRNAs (Fig. 5*D*). This rate is close to that observed for c-JUN (8%) while only 2% of the control mRNAs are retained.

Altogether these results show that the eIF3 subunits c, d, and g are capable of interacting with histones H1, H2A, H2B, H3, and H4 mRNAs *in vitro* independently of the eIF3 complex and confirm our cross-linking data. His-GSTeIF3e only interacts with H1 mRNA but not with H4 mRNA, unlike what was observed in our cross-linking experiments. It is likely that the interaction of eIF3e with H4 mRNA can only take place in the context of the entire eIF3 complex. Overall, our results reveal the existence of different interaction patterns between the eIF3 complex and the different histone mRNAs, this is consistent with previous observations concerning other eIF3 mRNA targets (19).

### Effect of the depletion of eIF3 c, d, e, and g subunits on histone mRNA binding and histone neosynthesis *in vivo*

To determine the importance of eIF3c, d, e, and g for histone mRNA binding and histone synthesis *in vivo*, we analyzed the effect of their depletion by siRNAs in HEK293 FT cells. Depletion of individual eIF3 subunits was performed for 48 h or 72 h using pools of four different nonoverlapping dual-strand modified siRNA to reduce potential off-target effects (38). The depletion levels of the target mRNAs and of the proteins were measured by qRT-PCR and western blot.

**Figure 6:**
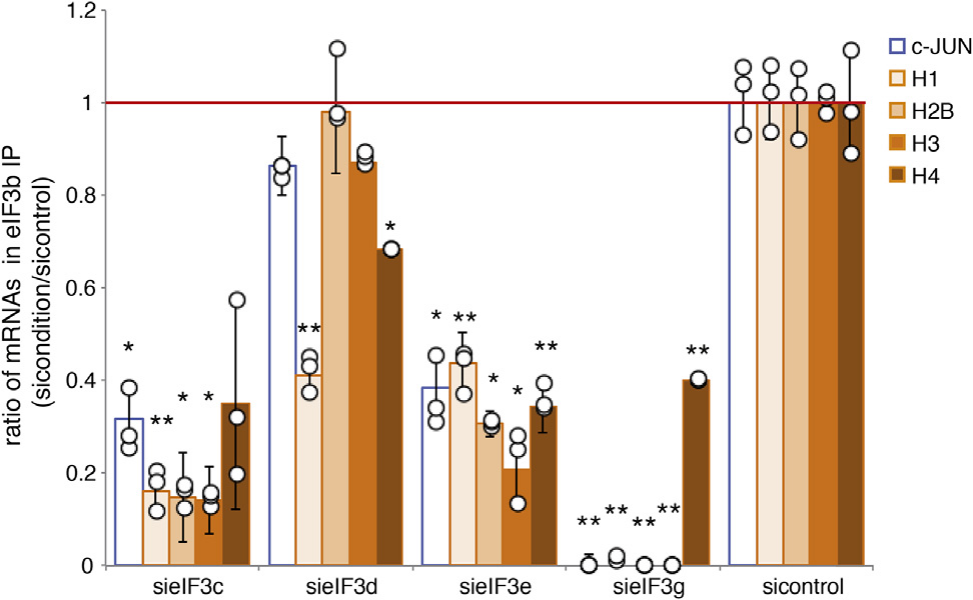
**Effect of the depletion of eIF3c, d, e, and g individual subunits on eIF3/mRNA binding efficiency.** Immunoprecipitation of the endogenous eIF3 complex in sieIF3c, d, e, and g inactivation conditions using antibodies against eIF3b (α-eIF3b) was performed as described in Figure 2. qRT-PCR analysis of the mRNAs interacting with the residual eIF3 complex was performed as described in Figure 2. The graph represents IP ratios between sieIF3c, d, e, or g and sicontrol conditions. *Circles* represent values obtained in independent experiments and bars correspond to the mean. Error bars represent standard deviation of three independent experiments. *Asterisks* indicate statistically significant differences with the sicontrol condition. ∗*p* < 0.05 and ∗∗*p* < 0.005 based on Student’s *t*-test. The *horizontal red line* represents the normalized mRNA binding level in the control condition.

Using eIF3b IP experiments we analyzed the ability of the depleted eIF3 complexes to bind mRNAs by qRT-PCR (Fig. 6). Because previous studies showed that RNAi knockdown of individual subunits could also impact the expression of other eIF3 subunits and alter the integrity of eIF3 (39, 40), we examined in parallel their effect on the levels of the other eIF3 subunits and on the integrity of the complex by immunoprecipitation and western blot (Table S1). We could recapitulate previously published observations. We showed that knockdown of eIF3c to 26% resulted in simultaneous downregulation of eIF3d, e, and i and partially altered the integrity of the complex (Table S1). Consistently, the mRNA-binding efficiencies of histone mRNAs and of the c-JUN mRNA positive control were reduced by 77% on average after eIF3c knockdown (Fig. 6). Depletion of eIF3e to 12% strongly reduced the level of eIF3d to 39%, as previously published (39), as well as weakly eIF3i to 73% but had no significant impact on the integrity of the rest of the complex (Table S1). It nevertheless reduced histone mRNA and c-JUN binding by 68% on average, revealing the importance of eIF3e for mRNA binding. Similarly, the depletion of eIF3g to 26% reduced the levels of eIF3c, e, and i. eIF3g depletion had the strongest effect on both eIF3 integrity (Table S1) and mRNA binding. It abolished c-JUN mRNA, H2B, and H3 mRNA interactions and strongly reduced H4 mRNA binding (Fig. 6). In contrast, and as previously published (39), the depletion of eIF3d to 55% affected neither the expression nor the integrity of eIF3 (Table S1). Depletion of eIF3d had the mildest effect on histone mRNA and c-JUN binding as only H1 and H4 mRNA binding was significantly reduced by 59% and 32% respectively (Fig. 6). These results confirm the importance of eIF3c, d, e, and g for histone mRNA binding but also eIF3 integrity. A clear correlation can be established between the level of integrity of the depleted eIF3 complexes and the level of histone mRNA binding *in vivo*.

**Figure 7:**
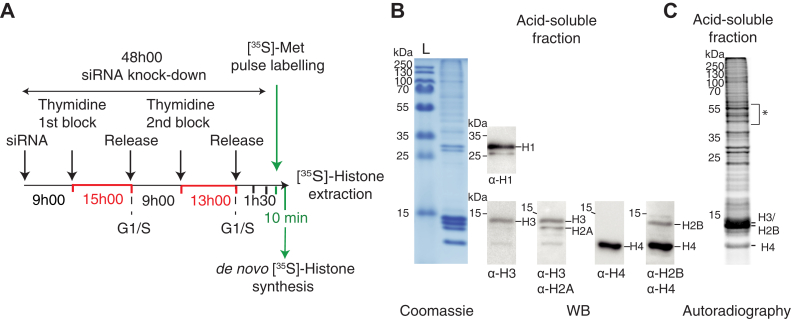
**Impact of the depletion of eIF3 subunits on histone neosynthesis.***A*, schematic of the methodology. siRNA-mediated depletion of eIF3 subunits was performed in HEK293FT cells for 48 h. Cells were synchronized at G1/S by a double thymidine block method during the siRNA inactivation phase for two periods of 15 h and 13 h separated by an interval of 9 h (marked in *red*) as indicated. Cells were released from blockage for 1 h and after 30 min of methionine starvation; *de novo* expression of proteins was followed by 10 min of [^35^S]-methionine pulse labeling. The depletion levels of the target mRNAs and of the proteins were verified by qRT-PCR and western blot (Fig. S1). The effects on *de novo* expression of total [^35^S]-proteins and neosynthesized [^35^S]-histones were monitored in parallel for each experiment. Neosynthesized [^35^S]-histones were selectively isolated from cell nuclei after precipitation of acid-soluble proteins (45), see also Fig. S2. *B*, fractionation of the resulting histones on SDS-PAGE and revelation by Coomassie staining and western blot using the indicated antibodies (α-H1, α-H2A, α-H2B, α-H3, and α-H4). To unambiguously identify the position of histone proteins, western blots were successively probed with a second antibody (case of α-H3/α-H2A and α-H4/α-H2B). The position of the molecular weight markers is indicated. (L) Ladder. Examples of results are shown in Fig. S2. *C*, autoradiography of the corresponding SDS-PAGE revealing only [^35^S]-neosynthesized histones. The levels of [^35^S]-histones were quantified using ImageQuant. Results were normalized against a group of nonhistone proteins designated by an *asterisk* (∗). Histones H2B and H3 comigrate and were quantified simultaneously.

**Figure 8:**
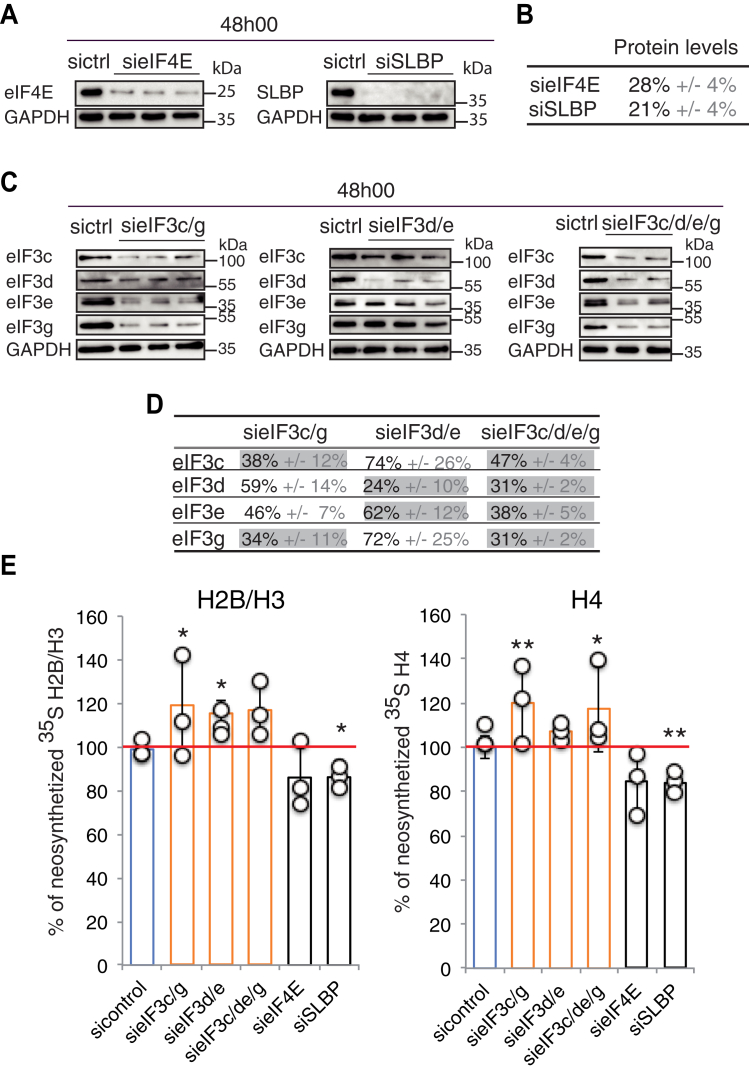
**Effect of depletion of multiple eIF3 subunits on histone neosynthesis.***A*, western blot analysis of the efficiency of siRNA knockdown on eIF4E and SLBP compared with sicontrol (sictrl). The position of the molecular weight markers is indicated. *B*, quantification of residual protein levels after 48 h of siRNA and measured by western blot. Normalization was performed against GAPDH. *C* and *D*, efficiency of double and quadruple eIF3 subunit depletions (eIF3 (c/g), eIF3 (d/e) and eIF3 (c/d/e/g) respectively) after 48 h of siRNA treatment analyzed by western blot. In panel *D* the expression levels of the targeted proteins are boxed in *gray*; the impact on the expression levels of all the four subunits eIF3c, g, d, and e is indicated. *E*, quantification of *de novo* [^35^S]-histone H2B/H3 and H4 synthesis as described in Figure 7. *Circles* represent values obtained in three independent experiments and the *bars* correspond to the mean. *Blue bars*, sicontrol; *orange bars*, siRNA against eIF3 subunits. Error bars represent standard deviation of an average of three independent experiments. *Asterisks* indicate statistically significant differences with the corresponding sicontrol conditions. ∗*p* < 0.05 and ∗∗*p* < 0.005 based on Student’s *t*-test. The *horizontal red line* represents the level of *de novo* histone synthesis in the control condition.

We next analyzed the effect of the four individual subunits depletion on histone translation. Histones are strongly and massively expressed only during the S phase of the cell cycle (41, 42). Cells were therefore synchronized at G1/S by the double thymidine block method (43) during the siRNA inactivation phase for two consecutive periods of 15 h and 13 h separated by an interval of 9 h (Fig. 7). As controls we knocked down the cap-binding factor eIF4E and the stem-loop binding protein (SLBP), a key factor for the processing of the 3’ end of histone mRNA (44). The cell synchronization in S phase was confirmed by flow cytometry after 1 h of blocking release (Fig. S1). At this stage, after 30 min of methionine starvation, *de novo* expression of proteins was followed by [^35^S]-methionine pulse labeling and neosynthesized [^35^S]-histones were selectively isolated from cell nuclei after precipitation of acid-soluble proteins (45). Proteins were resolved by SDS-PAGE, stained with Coomassie blue (Fig. 7*B*), and radiolabeled histones were quantified using a Phosphorimager and normalized against a group of discrete nonhistone proteins (Fig. 7*C*). This allowed the detection of H3, H2B, and H4, but not H1 and H2A, which lack internal methionine, the N-terminal methionine being most likely processed during protein biosynthesis. H2B and H3 were quantified simultaneously because they cannot be separated easily on the gel. Examples of results are shown in Fig. S2. Using a WST-1 metabolic assay we showed that the activity of mitochondrial dehydrogenases was not affected upon knock-down of the eIF3 subunits or SLBP, except for eIF3e and eIF4E for which the activity of dehydrogenases was reduced by 20% after 48 h of siRNA knockdown (Fig. S1). Our siRNA conditions did not significantly alter the viability of the cells, thereby making it possible to analyze the impact of this depletion on histones neosynthesis. After 48 h of siRNA knockdown the target mRNA levels were all reduced below 20%. The corresponding levels of eIF3c, d, e, and g proteins could be lowered to 30% on average, that of eIF4E and SLBP to 28% and 21% respectively, while the level of the control protein GAPDH remained unchanged (Fig. 8 and Fig. S3, *A* and *B*). The strong inactivation of the control protein SLBP resulted in a statistically significant decrease of 15 and 17% of histones H2B/H3 and H4 translation (Fig. 8). These rather modest effects on translation are similar to those obtained by others (46) for the inactivation of SLBP in U2OS cells and are likely due to residual proteins present in the cells. The depletion of eIF4E also decreased the translation of H2B/H3 and H4 by 15% while in the same conditions siRNA-mediated depletion of eIF4E can lead to a maximum of 25% downregulation of the translation of a canonical reporter gene (47). No effect was observed in samples treated with control siRNAs (Fig. 8). Under these conditions, the depletion of different individual eIF3 subunits seems to have modest and not always significant effects on histone translation (Fig. S3*C*) when compared with their impact on histone mRNA binding (Fig. 7). This is the case for eIF3d, e, and g depletion that nevertheless slightly increased the level of translation of H2B/H3 and H4 (Fig. S3*C*). On the contrary, eIF3c knockdown reduced the expression levels of histones H2B/H3 and H4, recapitulating SLBP or eIF4E depletion effects (Fig. S3*C*). These effects were measured under conditions of early depletion in order to minimize the inevitable impact on the integrity of the endogenous eIF3 complex (Table S1). Because of these limitations, it is unclear how individual eIF3 subunits contribute to histone mRNA translation. To overcome these problems and simulate a more global eIF3 complex downregulation, we inactivated simultaneously two or four subunits of eIF3 (eIF3c/g, eIF3d/e, or eIF3c/d/e/g) and analyzed histone neosynthesis after 48 h (early depletion) (Fig. 8). Results show that after 48 h of siRNA the expression levels of the targeted subunits can be reduced down to 35% in average. Simultaneous depletion of eIF3c and g also indirectly impacted the levels of eIF3d and eIF3e (Fig. 8, *C* and *D*) as previously reported (40) and strongly impacted eIF3 complex formation as revealed by immunoprecipitation and western blot analysis (Table S1). Surprisingly, after 48 h the effects of multiple eIF3 subunit inactivation lead to a statistically significant 20% increase of histones neosynthesis compared with global protein synthesis or to GAPDH (Fig. 8*E*). These results suggest that destabilization of the eIF3 complex can selectively modulate histone translation and that eIF3 can act as a negative regulator of histone synthesis. In an attempt to amplify the effects we continued multiple subunit silencing for an additional 24 h. In these conditions the targeted proteins could be further reduced but we measured a global reduction of cellular fitness and disassembly of the entire eIF3 complex (Fig. S4, *A*–*C*). As a result, the neosynthesis of histones H2B/H3 and H4 dropped by 50% compared with nonhistone control proteins (Fig. S4*D*). Similar downregulation was obtained after 72 h for the single eIF3e subunit silencing (48). Altogether, these data indicate that eIF3 plays a direct role in the translation control of histone mRNAs.

## Discussion

Histones mRNAs combine several specific features ensuring efficient translation during the S phase. These cell-cycle-dependent mRNAs are derived from intronless genes, have usually small 5’UTR, and are deprived of poly(A) tails. Instead, these mRNAs include in their 3’ end a highly conserved 16 nt hairpin structure interacting with the SLBP that plays key roles in their processing, export, and translational activity (44, 49, 50, 51). In metazoan cells, histones are massively and exclusively expressed during the S phase of the cell cycle. In addition, histone mRNAs are only detected in mid-S phase for about 1 h, but 60 million copies per core histones must be synthesized during this time lapse thanks to a highly productive translation mechanism (52). Histone H4 mRNA contains structural elements critical for efficient translation initiation. A double stem-loop structure called eIF4E sensitive element (4E-SE) binds eIF4E without the need of the cap and ribosomal 43S particles become tethered to this site. This allows direct loading of the 43S in the vicinity of the AUG. Another structure, located downstream of the initiation codon, forms a TWJ, which sequesters the m^7^G cap. This element facilitates direct positioning of the ribosome on the cognate start codon. Translation initiation of histone H4 can thus be considered as a hybrid mechanism between canonical and IRES-driven translation initiation (17). The lack of scanning appears to favor high expression levels of histone H4 during the S phase. Using structure probing in solution we have identified an additional stem-loop structure in the coding region of H4 mRNA located between the 4E-SE and the UAA stop codon. The function of this structure remained elusive until PAR-CLIP experiments revealed the presence of a putative binding site for the initiation factor eIF3 in its 3’ strand (19). Here we have shown that eIF3 binds directly to this H4 mRNA stem-loop structure called hereafter eIF3-BS (eIF3 binding site). Binding sites of eIF3 were predominantly mapped to the 5’UTR or the 3’UTR (19). The eIF3-BS of H4 mRNA is so far the first example of eIF3 binding site that is located in the coding region of an mRNA and adjacent to structural elements that facilitate the recruitment of the translation initiation machinery. These results expand the model of H4 mRNA translation to include an eIF3-dependent regulation mechanism.

We have demonstrated that binding of eIF3 to H4 mRNA is mediated by the subunits eIF3c, d, e, and g that cross-link to H4 mRNA. We confirmed the existence of direct interactions between the subunits c, d, g, and H4 mRNA. Mammalian eIF3 is composed of two interconnected modules assembled around the eIF3a/eIF3b nucleation core (11, 23, 24, 39). The subunits of eIF3 in contact with H4 mRNA are located in both modules. While the PCI subunits eIF3c and e are part of the octameric subunits and are positioned near the mRNA exit channel (24), eIF3g belongs to the b-g-i-a-CTD Yeast Like Core subcomplex and is in contact with the mRNA entry channel (27, 53). eIF3d is located on the eIF3 periphery and is attached to the octamer through eIF3e (53, 54) but also interacts with eIF3c and probably eIF3a (24). Among these subunits some contain previously characterized RNA-binding activities, interacting with IRES structures for instance. eIF3c contains a RNA-binding helix-loop-helix (HLH) motif (55) that interacts with rRNA on the back of 40S (24) and the PCI domain interacts with RNA to promote assembly of translation preinitiation complexes (56). eIF3g contains an RRM (57) while eIF3d is capable of binding to the cap of various mRNAs (20). The RNA-binding HLH motif identified in eIF3c was shown to contribute to HCV IRES binding (55). Deletions of amino acids 302 to 343 from subunit c, which include the HLH motif, reduced the apparent affinity of the eIF3 octameric core for the IRES over 100-fold compared with wild-type octamer. Our data show that the histone mRNA-binding domain is located between amino acids 1 and 318 of eIF3c. Binding sites for these different RNA substrates of eIF3c thus partially overlap. Nevertheless, the complete HLH structural motif is absent from recombinant eIF3c 1 to 318 proteins that contain only the N-terminal helix of the HLH motif. HCV IRES–eIF3 interaction has been proposed to substitute for translation initiation factor eIF4G, which is required for cap-dependent translation and to rely on direct contacts with eIF3a/c or b/c subunits (1, 32, 33, 58). We did not identify cross-links with eIF3a nor b. Altogether this suggests a different mode of interaction of the eIF3 complex with H4 mRNA. Furthermore our results also reveal an interaction pattern different from that observed by PAR-CLIP for most cellular mRNAs that involve distinct combinations of eIF3a, b, d, and g subunits (19). Additionally, we showed that the interaction pattern of eIF3 also varies between different histone mRNA targets. In particular, it appears that the isolated eIF3e subunit interacts directly only with histone H1 mRNA but not with the other histone mRNAs tested and that eIF3g also shows strong preference for H1 mRNA. eIF3d and g interact with histone H1, H2A, H2B, H3, and H4 mRNAs independently of the eIF3 complex. Interestingly eIF3e and eIF3g play specific roles in histone mRNA translation (48, 59) and eIF3e was shown to form with eIF3d a module that orchestrates the expression of specific mRNAs involved in the control of cellular metabolism (60). Altogether our results suggest that interactions of histone mRNAs with eIF3 rely on unique functional patterns and that distinct modes of interactions can exist between eIF3 and the RNAs it controls.

Our functional analysis showed that silencing of individual eIF3 subunits *in vivo* has very moderate but also dual effects. This does not allow drawing a clear picture of the contribution of individual eIF3 subunits to the translation of histone mRNAs. Depletion of eIF3 d, e, and g only mildly increased histone neosynthesis while depletion of eIF3c had the opposite effect and decreased histone synthesis. Such dual and varying effects have been reported in previous downregulation studies depending on whether eIF3a, eIF3c, eIF3e, or eIF3j subunits were inhibited (39, 40, 48). Functional redundancy between eIF3 subunits has been proposed. In particular, depletion of eIF3c *in vivo* by siRNA leads to the appearance of an eIF3a, b, g, and i subcomplex (resembling the minimal eIF3 “Yeast Like Core”), which retained a high affinity for the 40S ribosomal subunit but with a relaxed specificity of recognition for the initiating AUG (40). In yeast, a complex composed only of eIF3a, eIF3b, and eIF3c was shown to stimulate translation initiation, possibly through interactions with eIF5 and eIF1 (12, 61), which also suggests that eIF3 components and other initiation factors can functionally compensate for each other. Multiple eIF3 subunit silencing results in the destabilization of the eIF3 complex and therefore provides a view of the global function of this complex in histone synthesis under early inhibition conditions. Our results suggest that decreasing the amount of eIF3 complex capable to bind histone mRNAs in a transcript-specific manner initially promotes the synthesis of histones and that eIF3 therefore acts as a repressor of histone mRNA expression. The effects observed are still milder than expected. eIF3 should therefore rather be considered as a modulator than as an inhibitor of histone synthesis. Long-term depletion of the eIF3 complex has a general deleterious effect on protein translation; this effect is even stronger for the translation of histones that are direct eIF3 mRNA targets. This confirms the crucial and specific role of eIF3 for the massive production of histones during the S phase. Overall, our interpretation is that, by binding to histone mRNAs, eIF3 can act as a translational modulator. At high concentrations of eIF3 this would limit the synthesis of histones that would otherwise be toxic to the cell (62). Under metabolic conditions where eIF3 would be limiting the production of histones would be favored. Several studies have shown that eIF3 subunits can have different expression patterns throughout the cell cycle (63, 64). For instance, eIF3 was found to bind *PTBP1* mRNA isoforms in a cell-cycle-dependent manner. A strong correlation could be established between eIF3 binding to *PTBP1* mRNAs and repression of PTBP1 expression during the S phase of the cell cycle (65). The translation of several additional mRNAs is repressed by eIF3. This is the case for the cell proliferation regulator BTG1 (19), ferritin light chain (*FTL*) mRNA (21), and msl-2 mRNA in *Drosophila melanogaster* (66). How eIF3 binding can contribute to the negative translation regulation of an mRNA and modulation of histone translational regulation in particular is unclear at this stage. The eIF3 complex could either bind alone to the mRNA or as part of ribosomal 43S particles. Interestingly the eIF3-BS is adjacent to two important structural elements: the 4E-SE element that recruits eIF4F and subsequently favors 43S particle tethering and a conserved stem loop (SL) in the 3’UTR that binds SLBP and is required for histone mRNA processing. In mammals, interactions between the eIF4F-mRNA complex and the 43S-PIC are stabilized by direct interactions between eIF4G, eIF4A, and eIF3. The eIF4G-binding surface in eIF3 was shown by biochemical cross-linking to precisely comprise the eIF3c, d, and e subunits (24, 67). Recent cryo-EM structure of the human 48S translational initiation complex also revealed that eIF4A interacts with the 43S-PIC through the eIF3 subunits eIF3e, k, and l (24). eIF3 binding to eIF3-BS could possibly hinder the recruitment of 43S particles. Interestingly eIF3e and eIF3g play specific roles in histone mRNA translation and promote the interaction between SLIP1 and SLBP that is necessary for efficient histone mRNA translation (48, 59). The eIF3g-SLIP1/SLIP1-SLBP can be compared with eIF3g-PAIP1-PABP interactions that contribute to the circularization of canonical mRNAs (68). The direct interactions of eIF3 with eIF3-BS could similarly hinder these interactions. Such mechanisms would be sensitive to the levels of eIF3 in the cells. Changes in eIF3 activity are correlated with several human disorders and altered levels of eIF3 subunits are associated with a variety of human cancers as many mRNAs controlled by eIF3 are associated with cell growth (69, 70, 71, 72). Here we show that eIF3 also contributes to regulatory mechanisms that coordinate the rates of histone synthesis. Altogether our results provide new insight into the mechanism of eIF3 selective translation regulation and expand our understanding of H4 mRNA translation.

## Experimental procedures

### Cell culture

HEK293FT cells were cultured at 37 °C in 5% CO_2_ in Dulbecco’s Modified Eagle Media (DMEM) containing 10% fetal calf serum, 1% penicillin-streptomycin (5000 U/ml penicillin, 5000 μg/ml streptomycin, Invitrogen), 500 μg/ml geneticin. Cells were extracted with RNP buffer (10 mM HEPES-NaOH pH 7.9, 100 mM KCl, 5 mM MgCl_2_, 0.5% NP-40, 1 mM DDT, 100 U/ml RNasin (Promega), 400 μM VRC (Vanadyl Ribonucleotide Complex Sigma), anti-protease cocktail from Sigma). To stabilize RNP complexes, formaldehyde cross-linking was performed. Cells were washed with DPBS (Gibco), centrifuged for 10 min à 4 °C, and the pellets were resuspended in 1 vol of 0.2% formaldehyde for 5 min. Cross-linking reactions were quenched by the addition of 0.15 M glycine pH 7 for 5 min. Cells were subsequently extracted in RNP buffer.

### Immunopurification and western blotting

Immunopurification of endogenous eIF3 complexes was performed in HEK293FT cells as described in (19, 73). In total, 300 μl of cell extracts was incubated in the presence of 2 μg of antibody directed against eIF3b (Bethyl, A301-761A) and 100 μl of protein A μMACS magnetic beads (Miltenyi) in 1 ml Lysis Buffer (50 mM Tris-HCl pH 8, 150 mM NaCl, 1% Triton X-100). Beads were washed four times according to the manufacturer’s instructions and eluted in Laemmli buffer. Proteins were analyzed by SDS-PAGE followed by western blot. Antibodies used are listed in Table S2. Bound RNA was extracted by phenol/chloroform and precipitated. After DNase treatment, RNAs were reversed transcribed using AMV-RT (Q-Biogen) and cDNAs were amplified by quantitative RT-PCR (qRT-PCR). Reactions were carried out on a CFX96 Real-Time PCR detection system (Bio-Rad) using the Maxima SYBR Green PCR kit (Thermo Scientific). Oligonucleotides used for qRT-PCR are listed in Table S3. The % of RNAs in IP was determined by the ΔCq method and normalized by the input RNAs. Results were expressed as mean ± standard error of an average of three measurements.

### Recombinant proteins and GST pull-down assays

Recombinant HisGST-tagged eIF3c truncated proteins: HisGSTeIF3d, HisGSTeIF3e, and HisGSTeIF3g were obtained from *E. coli* by standard procedure and purified using Ni-NTA agarose (Qiagen). For GST pull-down experiments purified HisGST proteins (40 μg) were bound to 50 μl of GST-Trap agarose beads (Chromotek) and incubated with 60 μg of HEK293FT total RNA in binding buffer (20 mM Tris-HCl pH 7.5, 100 mM KCl, 0.1 mM EDTA, 1 mM DTT, 10% glycerol, 400 μM VRC, 100 U RNasin/ml, anti-protease cocktail) for 30 min at 4 °C. Beads were washed three times with binding buffer. The RNAs present in the flow-through or on the beads were extracted by phenol/chloroform, precipitated and quantified by qRT-PCR as previously described.

### RNA probing

The transcript of entire H4 mRNA was probed by selective 2’-hydroxyl acylation analyzed by primer extension (SHAPE) and chemical modification of the bases with the chemical dimethyl sulfate (DMS) and 1-cyclohexyl-3-(2-morpholinoethyl)carbodiimide metho-p-toluene sulfonate (CMCT). The sites of chemical modification were subsequently defined by a primer extension-termination assay in which cDNA was synthesized by reverse transcriptase from a fluorescent-complementary oligonucleotide, which was hybridized to nt 357 to 376 downstream of the region of interest.

Prior modification, the H4 mRNA transcript was heated 2 min at 95 °C and placed on ice for 2 min. SHAPE modification was performed in 10 μl containing 2 pmoles RNA (0.5 μM final concentration), 80 mM benzoyl cyanide, 10% DMSO, 90 mM Na HEPES, pH 8.0. After 10 min at 20 °C, the modified RNA was precipitated. DMS probing was performed as follow: 2 pmoles of the H4 mRNA were incubated for 10 min in 20 μl DMS buffer (50 mM Na cacodylate, pH 7.5, 5 mM MgCl_2_, 100 mM KCl), 0.05 mg/ml total tRNA. Then, the RNA was modified in the presence of 1.25% DMS for 10 min at 20 °C and terminated on ice. The modified RNA was precipitated with ethanol. CMCT probing was performed as follow: 2 pmoles of the H4 mRNA were incubated for 10 min in 20 μl CMCT buffer (50 mM Na borate, pH 8.5, 5 mM MgCl_2_, 100 mM KCl), 0.05 mg/ml total tRNA. Then, the RNA was modified by adding 5 μl of a solution 42 g/l CMCT. After 10 min at 20 °C, 2 μl 100% ethanol was added, and incubation was performed for another 10 min before terminating on ice. The modified RNA was precipitated with ethanol.

Chemical modifications were detected by primer extension with fluorescent primers complementary to the 3’ sequence of the mRNA. Reverse transcription was performed in 20 μl containing 2 pmoles RNA, 0.9 pmole of a fluorescently labeled primer (VIC or NED, from Integrated DNA Technologies), 160 U SuperScript III reverse transcriptase, 83 mM KCl, 56 mM Tris-HCl, pH 8.3, 0.56 mM each dNTP, 5.6 mM DTT, 3 mM MgCl_2_. The RNAs were first denatured at 95 °C for 2 min, followed by annealing at 65 °C for 5 min and incubation on ice for 2 min. RT extension was performed at 42 °C for 2 min, 50 °C for 30 min, and then 65 °C for 5 min. Sequencing reactions were performed in parallel in similar conditions, but containing 0.5 mM ddTTP. Reactions were stopped by the addition of 4 μl 50 mM EDTA pH 8.0, phenol-chloroform extracted, precipitated, washed, dried, and resuspended in 10 μl deionized formamide. Samples were loaded on a 96-well plate for sequencing on an Applied Biosystems 3130xl genetic analyzer. For each probing reagent, three experiments were performed in the presence or absence of the reagent. The resulting electropherograms were analyzed using QuSHAPE (74) as described (16).

### Electrophoretic mobility shift assay

Transcription templates for the synthesis of full-length H4 mRNA (1–375) and truncated H4 mRNAs (1–137, 137–241 and 241–375) were generated by PCR as described in (17). The 5’ primers contained the T7 promoter sequence, and the 3’ primers were designed to promote *in vitro* run-off transcription at the desired position. Internally labeled H4 mRNAs transcripts were obtained by *in vitro* transcription with T7 RNA polymerase using 50 μCi of [α-^32^P]-GTP (6000 Ci/mmol). Transcripts were purified by denaturing 4% PAGE and recovered by passive elution in Elution Buffer (10 mM Tris-HCl, pH 7.5, 0.3 M NaCl, 0.5 mM EDTA). Purified RNA samples were phenol extracted and ethanol precipitated. Before use, the H4 mRNA transcripts were heated 2 min at 95 °C and placed on ice for 2 min. H4 mRNA-eIF3 complexes were formed as described in (75). eIF3 complex purified from rabbit reticulocyte lysates was supplied by Prof. WC Merrick (76). For mobility shift assays, 15,000 cpm of [^32^P]-labeled H4 mRNAs was incubated for 30 min at 25 °C with increasing concentrations of eIF3 complex in 5 μl of binding buffer (25 mM Tris-HCl, pH 7.5, 5 mM Mg(OAc)_2_, 70 mM KCl, 0.1 mM CaCl_2_, 0.1 mg/ml BSA, 2 mM TCEP). After addition of 1 μl of 6X nondenaturing loading dye (40% sucrose, 0.025% xylène cyanol, 0.025% bromophenol blue) RNA–protein complexes were separated on 0.7% agarose gel in TBE, 75 mM KCl buffer. The gel was run for 2 h at 40 V at 4 °C and the buffer was replaced by fresh cold buffer after an hour. The gel was transfered to Hybond N+ (Amersham) nylon membrane for 3 h at 70 °C using a preheated dryer. The intensities of free and bound RNAs were quantitated by Phosphor imaging.

### *In vitro* transcription of 4-thioU and [α-^32^P]-ATP-labeled mRNAs

The synthesis of DNA templates was performed as described previously (77). RNAs were 4-thioU labeled during transcription using a 2:1 M ratio of 4-thioUTP:UTP. 5 mM of (CTP, GTP, and ThioU) (Jena Bioscience), 2.5 mM UTP and 10 μl of [α-^32^P]-ATP (6000 Ci/mmol) in TMSDT buffer (40 mM Tris-HCl pH 8, 1, 22 mM MgCl_2_, 1 mM spermidine, 5 mM DTT, 0.01% Triton X-100) in the presence of 40 U of RNasin and T7 RNA polymerase (0.2 mg/ml final) at 37 °C. Cold ATP was added five times every 10 min (1 μl at 50 mM) to reach a final concentration of 5 mM after 1 h. Pyrophosphatase (0.01 mg/ml) was added for 30 min and DNase I (20 U/ml final) was used to degrade the DNA template for 1 h 37 °C. RNAs were purified by phenol-chloroform extraction and ethanol precipitation followed by denaturing gel electrophoresis purification.

### ThioU H4 mRNA-eIF3 cross-linking reactions and 2D gel analysis

Radiolabeled ThioU-H4 mRNA (50,000 cpm) was incubated in the presence of 5 μM final of the purified eIF3 protein complex in a final volume of 4 μl of cross-linking buffer (100 mM KCl, 20 mM Tris HCl, pH 7.5, 1 mM DTT, 0.1 mM EDTA, 10% glycerol) for 30 min at 25 °C. The ThioU mRNA–protein cross-linking reaction was performed by UV 365 nm irradiation for 30 min. RNase A (Roche) digestion was carried out for 30 min at 37 °C in order to degrade mRNA fragments not protected by eIF3 subunits. Radioactive mRNA fragments cross-linked to eIF3 subunits remain bound to the proteins.

To identify eIF3 subunits interacting with H4 ThioU mRNA, cross-linking reactions were analyzed by 2D gel electrophoresis followed by western blot. To this end, cross-linking products were precipitated overnight at −20 °C with five volumes of ammonium acetate solution (0.1 M AcNH_4_, 100% methanol), centrifuged at 13,000 rpm (15,500*g*) for 15 min at 4 °C, and washed twice with (0.1 M AcNH_4_; 80% methanol). The pellets were dried and resuspended in 125 μl of UTCT buffer (7 M urea, 2 M thiourea, 4% CHAPS, 50 mM DTT, 0.2% ampholytes 3/10). Samples were separated by isoelectric focusing (IEF) in the first dimension (pH 4–7) using ReadyStripTM IPG Strips (BioRad) in a Protean IEF Cell generator (BioRad) according to the manufacturer’s conditions. The strips were successively equilibrated in equilibration buffer 1 (6 M urea, 0.375 M Tris-HCl, pH 8.8, 2% SDS, 20% glycerol, 2% DTT) to break the sulfhydryl groups for 10 min and equilibration buffer 2 (6 M urea, 0.375 M Tris-HCl, pH 8.8, 2% SDS, 20% glycerol, 2.5% iodoacetamide) for alkylation and reduction of sulfhydryl groups. Separation in the second dimension was performed on a 10% SDS-PAGE in TGS buffer (25 mM Tris-HCl, pH 8.8, 200 mM glycine, 0.1% SDS). Radiolabeled proteins were transferred to an Immobilon-P membrane (Millipore). The membrane was scanned by Phosphorimaging and subjected to western blot analysis using the ChemiDoc imaging system (BioRad); 2-D radioactivity and western blot images were superimposed.

### siRNA knockdown and protein synthesis analysis by pulse labeling

HEK293FT cells were used to analyze the effect of depletions of the four eIF3 subunits (c, d, e, and g), eIF4E and SLBP on *de novo* expression of histone proteins. Twenty-four hours before siRNA transfection, 4,10^5^ cells were cultured in six-well dishes. siRNAs consisting of pools of four different 2’-O-methylated siRNAs per target (ON-TARGET plus SMART pools, Dharmacon) were transfected with Lipofectamine 2000 (Invitrogen) following the manufacturer’s conditions. siRNAs are listed in Table S4. The simultaneous inactivation of two (eIF3 c/g, eIF3 d/e) or four targets (eIF3 c/d/e/g) was carried out in the presence of 100 pmole of each siRNA. For each condition three biological replicates were performed. Cell proliferation assays were performed using WST-1 (G-biosciences) to follow the effects of siRNA treatment on cell viability. The formazan dye (yellow) produced by cleavage of WST-1 in metabolically active cells was quantified using a multiwell spectrophotometer at 450 nm, 30 min after WST-1 addition. The analysis of the effect of siRNA knockdown on histone *de novo* synthesis was performed after 48 and 72 h. Histones are massively expressed during the S phase of the cell cycle. HEK293FT cells were therefore synchronized concomitantly at G1/S by the double thymidine block method as described by (43). After 9 h of siRNA transfection cells were blocked by addition of 2 mM thymidine (Sigma-Aldrich) for 15 h. Thymidine was removed, cells were rinsed with DMEM/10% FBS medium and incubated in 1 ml standard medium for 9 h. A second 2 mM thymidine block was performed for 13 h to obtain cells in G1/S transition. Cell synchronization was verified by flow cytometry and Fluorescence Activated Cell Sorting (FACS). The cells were released from the blockage and incubated for 1 h in DMEM/10% FBS medium. To follow *de novo* protein translation, cells were washed with Met-free starvation medium (DMEM Glutamax Gibco) as described in (78). The medium was replaced with Met-free DMEM supplemented with 100 μCi/ml [^35^S]-methionine and labeled for 10 min. The radioactive medium was removed and cells were washed three times with cold PBS. 25% of the cells were lysed in RNP buffer to prepare total cellular extracts, while the remaining 75% were used to extract the neosynthesized histones (45). The cell pellet was lysed in a nuclear isolation buffer (250 mM sucrose, 1 mM CaCl_2_, 2 mM MgCl_2_, 1% Triton X-100, 10 mM Tris-HCl, pH 8) for 1 h on ice. Nuclei were pelleted at 5000 rpm for 5 min at 4 °C. The nuclear pellet was resuspended in 0.4 N HCl and incubated on ice for 30 min. Nuclear samples were centrifuged at 15,000 rpm for 15 min at 4 °C and acid-soluble proteins precipitated in 20% trichloroacetic acid at −20 °C overnight. Samples were then centrifuged at 15,000 rpm for 30 min at 4 °C. Protein pellets were washed three times with cold acetone, dried, and resuspended in 20 μl of RIPA lysis buffer (150 mM NaCl, 5 mM EDTA pH 8, 50 mM Tris-HCl pH 8, 1% NP-40, 0.5% sodium deoxycholate, 0.1% SDS) supplemented with a cocktail of protease inhibitors (Sigma). Samples were analyzed by SDS-PAGE and autoradiography. Histones were quantified using ImageQuant software. In total cellular extracts the amount of [^35^S]-labeled protein was quantified after TCA precipitation using a scintillation counter (LS-6500, Beckman CoulterTM).

## Data availability

All the data are contained within the article. Supporting information is available online.

## Supporting information

This article contains supporting information.

## Conflict of interest

The authors declare no conflicts of interest in regard to this article.
