# Supplementary material for: eIF3 interacts with histone H4 messenger RNA to regulate its translation
Source: J Biol Chem. 2021 Mar 23;296:100578. doi: 10.1016/j.jbc.2021.100578 (PMC8102920; doi:10.1016/j.jbc.2021.100578)
Supplement: Supplemental Figures S1–S4 and Tables S1–S4 [file mmc1.pdf]

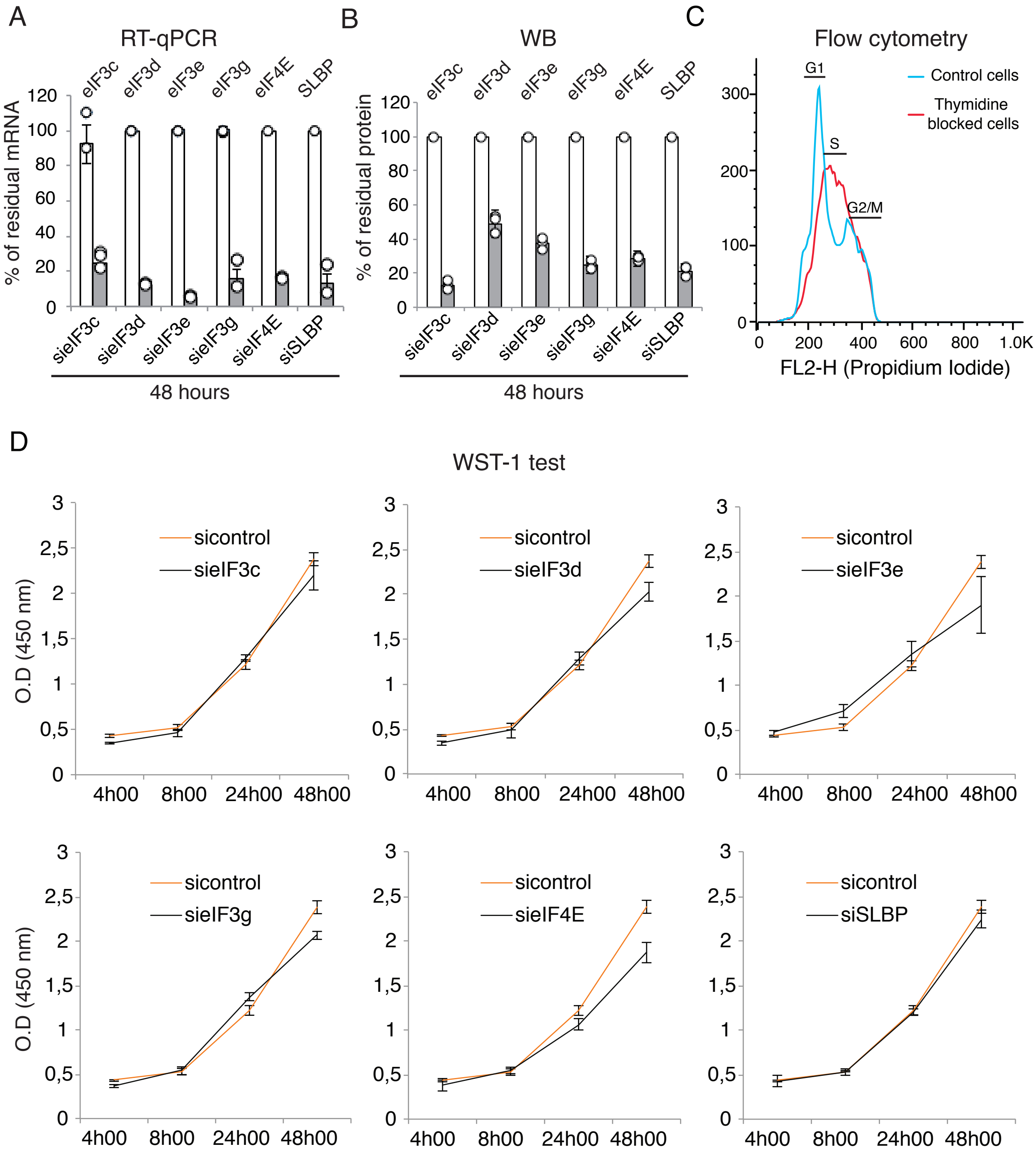

Figure S1: Measure of the efficiency of the knockdown of eIF3 c, d, e, and g subunits and their impact on cell viability. (**A**) Quantification of mRNA levels by qRT-PCR after siRNA treatment (grey bars). The white bars represent the sicontrol conditions. (**B**) Quantification of the residual proteins by western blot (WB) for the 4 subunits of eIF3c, d, e and g, as well as for the two controls eIF4E and SLBP. In panels A and B the graphs represent values obtained in three independent experiments (white circles) and bars correspond to the mean +/- SD. (**C**) Flow cytometry analysis of HEK293FT to validate cell synchronization in G1/S. The graph shows results for HEK293FT cells after double thymidine block (in red) compared to untreated cells (in blue). The DNA intercalating fluorescent dye propidium iodide is used to quantify cells in G1, S and G2 / M. FL2-H represents the intensity of the fluorescence signal at 617 nm. (**D**) Cell proliferation assay: measurement of the activity of mitochondrial dehydrogenases using the WST-1 reagent after 4, 8, 24 and 48 h of knockdown of the 4 subunits of eIF3 (c, d, e and g) as well as the two control proteins eIF4E and SLBP. The results are compared with those obtained with control siRNAs.

A

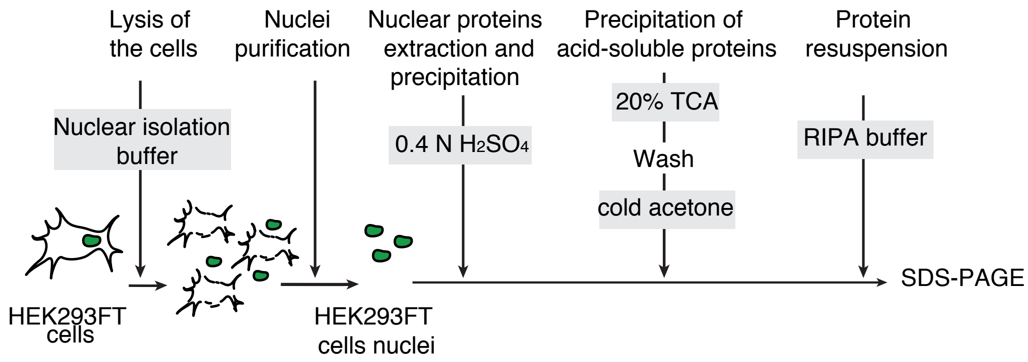

B

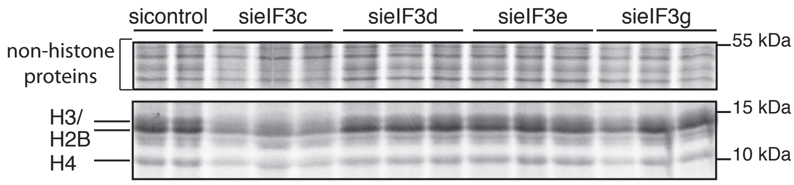

	sicontrol	sieIF3c	sieIF3d	sieIF3e	sieIF3g
H3/H2B	100	77 ± 5	110 ± 12	112 ± 12	123 ± 13
H4	100	85 ± 10	105 ± 11	137 ± 13	129 ± 11

C

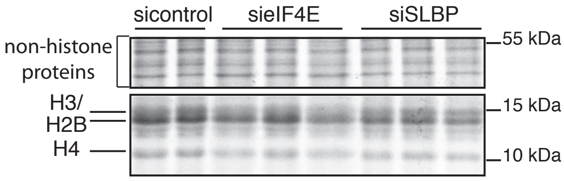

	sicontrol	sieIF4E	siSLBP
H3/H2B	100	86 ± 9	86 ±4
H4	100	84 ± 6	83 ± 3

D

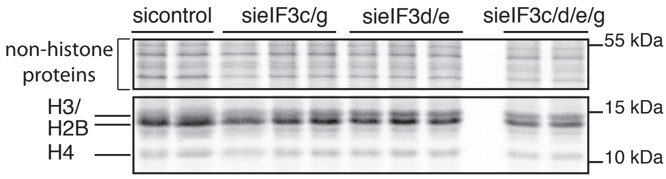

	sicontrol	sieIF3c/g	sieIF3d/g	sieIF3c/d/e/g
H3/H2B	100	120 ± 15	115 ± 5	132 ± 18
H4	100	119 ± 10	105 ± 4	122 ± 10

Figure S2: (**A**) Experimental design of the histone purification method. Acid-soluble histones are extracted from purified HEK293FT nuclei in 0.4 N H_2SO_4 and precipitated in 20 % trichloroacetic acid (TCA). The pellets containing the histones are washed with cold acetone and suspended in RIPA buffer before being analyzed by SDS-PAGE. (**B**-**D**) Examples of autoradiographs showing *de novo* synthesis of [^{35}S]-histones after 48h of eIF3 subunit knockdowns and separation on SDS-PAGE. The position of the molecular weight markers is indicated. (**B**) Results of eIF3c, d, e and g inactivation. (**C**) Inactivation of the control proteins eIF4E and SLBP. (**D**) Double and quadruple knockdowns of the eIF3 subunits (eIF3c/g, eIF3d/e and eIF3c/d/e/g). The levels of [^{35}S]-histones were quantified using ImageQuant. Results were normalized against a group of non-histone proteins. Histones H2B and H3 co-migrate and were quantified simultaneously.

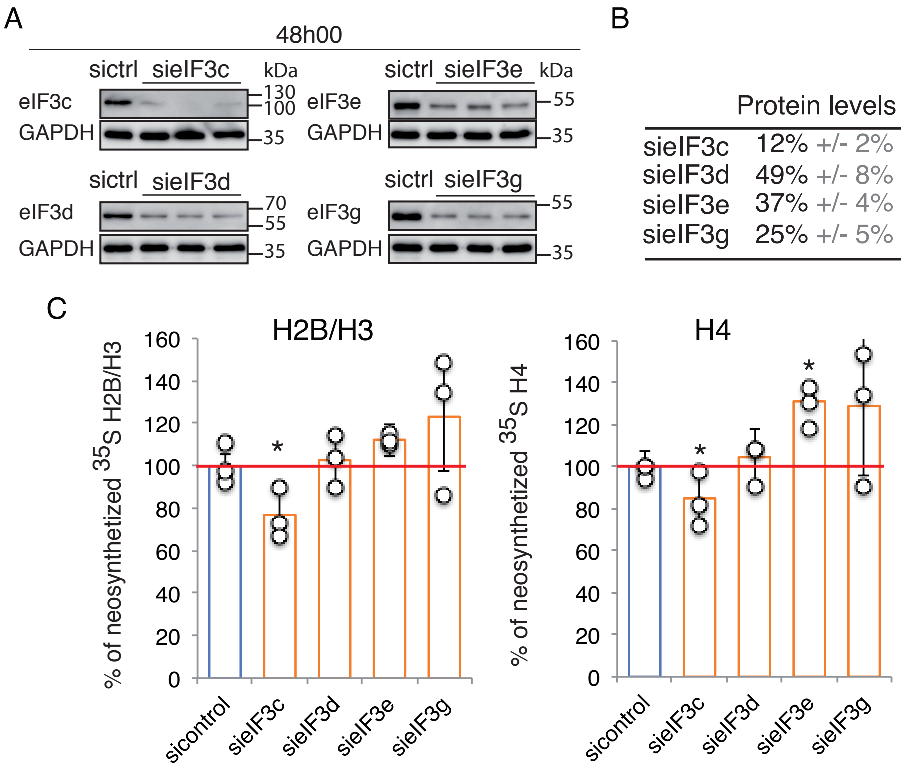

Figure S3: Effect of the depletion of individual eIF3 subunits c, d, e and g on the neosynthesis of histones. (**A**) Western blot analysis of the efficiency of siRNA knockdown on eIF3 c, d, e, g subunits compared to sicontrol (sictrl). The position of the molecular weight markers is indicated. (**B**) Quantification of residual protein levels after 48 h of siRNA and measured by western blot. Normalization was performed against GAPDH. (**C**) Quantification of *de novo* [^{35}S]-histone H2B/H3 and H4 using Image-Quant as described in Figure 7. Circles represent values obtained in 3 independent experiments and the bars correspond to the mean. Blue bars: sicontrol; orange bars: siRNA against eIF3 subunits. Error bars represent standard deviation of an average of 3 independent experiments. Asterisks indicate statistically significant differences with the corresponding sicontrol conditions. *$p < 0.05$ and **$p < 0.005$ based on Student's t test. The horizontal red line represents the level of *de novo* histone synthesis in the control condition. After 48 h of siRNA knockdown the levels of eIF3c, d, e and g proteins could be lowered to 12 %, 49 %, 37 % and 25 % respectively, while the level of the control protein GAPDH remained unchanged. As a result, the depletion of eIF3c subunit significantly reduced the expression levels of histones H2B/H3 and H4 by 23 % and 15 % respectively. The depletion of the subunits d, e and g, on the contrary, slightly increased the level of translation of H2B/H3 and H4 but only depletion of eIF3e lead to a significant 37 % increase of H4 expression compared to the non-histone internal control proteins.

A

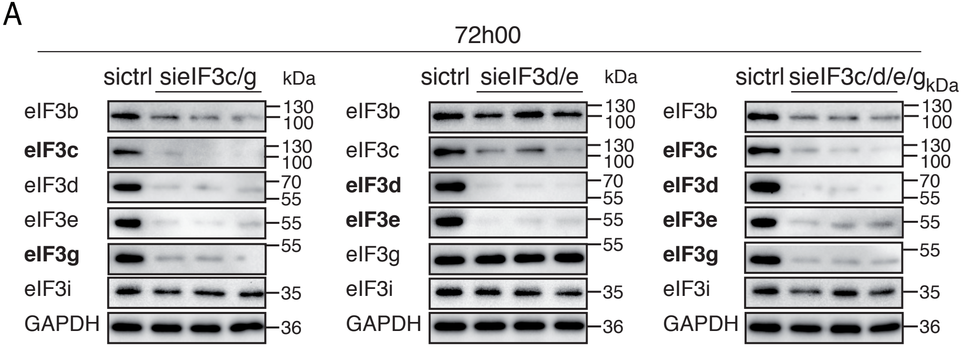

B

	sieIF3c/g	sieIF3d/e	sieIF3c/d/e/g
eIF3b	50% +/- 8%	79% +/- 9%	53% +/- 5%
eIF3c	14% +/- 6%	30% +/- 9%	20% +/- 5%
eIF3d	20% +/- 3%	10% +/- 3%	12% +/- 3%
eIF3e	24% +/- 6%	20% +/- 2%	55% +/- 13%
eIF3g	16% +/- 5%	129% +/- 19%	25% +/- 4%
eIF3i	89% +/- 8%	91% +/- 8%	68% +/- 9%

C

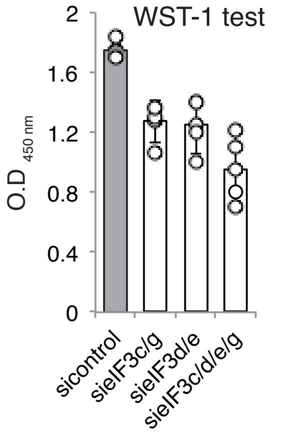

D

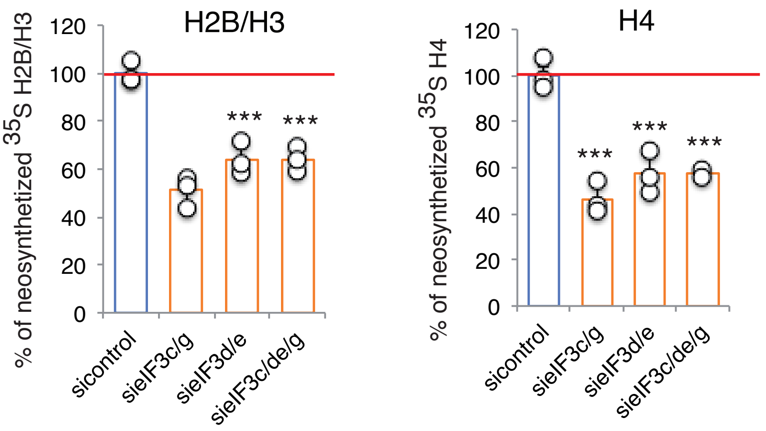

Figure S4: Effect of 72 h depletion of multiple eIF3 subunits on the neosynthesis of histones. (**A**) Western blot analysis of the efficiency of siRNA knockdown on eIF3 c/g, d/e and c/d/g/e subunits. (**B**) Quantification of residual protein levels after 72 h of siRNA and measured by western blot. Normalization was performed against GAPDH. The expression levels of the targeted proteins are boxed in grey. (**C**) WST-1 metabolic assay (G-biosciences) measuring the activity of mitochondrial dehydrogenases. Results were obtained from 5 independent measurements (**D**) Quantification of *de novo* [^{35}S]-histone H2B/H3 and H4 synthesis using Image-Quant. Values obtained in 3 independent experiments are represented by white circles and the bars correspond to the mean. Blue bars: sicontrol; orange bars: siRNA against eIF3 subunits. Error bars represent the standard deviation. Asterisks indicate statistically significant differences with the corresponding sicontrol conditions. *$p < 0.05$ and **$p < 0.005$ based on Student's t test. After 72 h of double or quadruple eIF3 subunit knockdowns the residual expression levels of the targeted proteins could be reduced to 15 % but the levels of other eIF3 subunits were also indirectly impacted. Cells therefore contain severely disrupted eIF3 complexes. The metabolic activity of the cells was reduced by 30 to 40 % compared to the control (grey bar). As a result the neosynthesis of histones H2B/H3 and H4 dropped by 40 to 50 %.

	sicontrol		sieIF3c		sieIF3d		sieIF3e		sieIF3g	
	WCE	eIF3b IP	WCE	eIF3b IP	WCE	eIF3b IP	WCE	eIF3b IP	WCE	eIF3b IP
eIF3b	100	100	83 ± 6	100	53 ± 4	100	157 ± 4	100	115 ± 3	100
eIF3a	100	100	193 ± 2	93 ± 5	117 ± 25	124 ± 5	146 ± 3	86 ± 3	250 ± 25	65 ± 16
eIF3c	100	100	26 ± 16	45 ± 10	100 ± 38	121 ± 40	138 ± 15	123 ± 15	78 ± 15	54 ± 6
eIF3d	100	100	60 ± 2	62 ± 23	55 ± 9	78 ± 3	39 ± 14	83 ± 5	184 ± 10	34 ± 15
eIF3e	100	100	63 ± 25	n.d	102 ± 24	n.d	12 ± 2	n.d	82 ± 14	45 ± 20
eIF3g	100	100	152 ± 4	n.d	105 ± 8	n.d	118 ± 29	133 ± 40	26 ± 10	33 ± 13
eIF3i	100	100	64 ± 2	51 ± 5	109 ± 4	171 ± 4	73 ± 7	247 ± 4	53 ± 5	64 ± 9

	sieIF3c,g		sieIF3d,e		sieIF3c,d,e,g	
	WCE	eIF3b IP	WCE	eIF3b IP	WCE	eIF3b IP
eIF3b	81 ± 8	100	121 ± 40	100	99 ± 33	100
eIF3a	49 ± 3	56 ± 5	114 ± 16	39 ± 3	52 ± 17	22 ± 33
eIF3c	49 ± 4	76 ± 2	53 ± 18	35 ± 2	21 ± 6	35 ± 29
eIF3d	44 ± 8	22 ± 1	28 ± 8	33 ± 1	18 ± 8	25 ± 25
eIF3e	23 ± 4	60 ± 2	19 ± 8	36 ± 5	26 ± 5	n.d
eIF3g	51 ± 3	0 ± 0	86 ± 40	43 ± 5	21 ± 6	n.d
eIF3i	14 ± 3	12 ± 1	118 ± 30	33 ± 4	34 ± 4	0 ± 0

Table S1: Quantification of anti-eIF3b co-immunoprecipitation experiments performed after siRNA inhibition of eIF3 c, d, e, g subunits in HEK293FT cells. The levels of endogenous eIF3 subunits after 48 h of siRNA were measured by western blotting, quantified in whole cell extracts (WCE) using Image Lab (BioRad) and normalized against GAPDH. To assess the integrity of the eIF3 complex, immunoprecipitations were performed for each knock-down using antibodies against eIF3b (eIF3b IP). The immunoprecipitated proteins were analyzed by SDS-PAGE, western blotting and quantification. Results were normalized against the bait eIF3b. Ratios are represented as percentages of the sicontrol experiment. The average of 3 independent experiments is shown ± SD. The inhibited subunits are highlighted in grey. n.d: not determined.

Table S2: List of antibodies for western blot or immunoprecipitation

Antibody	Origin	Reference
**Anti-eIF3a**	Polyclonal /Rabbit	Novus Biological / NBP1-18891
**Anti-eIF3b**	Polyclonal / Rabbit	Bethyl / A301-761A
**Anti-eIF3c**	Polyclonal / Rabbit	Bethyl / A300-377A-T
**Anti-eIF3d**	Polyclonal / Rabbit	Bethyl / A301-758A
**Anti-eIF3e**	Polyclonal / Rabbit	Bethyl / A302-985A
**Anti-eIF3f**	Polyclonal / Rabbit	Bethyl / A303-005A
**Anti-eIF3g**	Polyclonal / Rabbit	Bethyl / A301-757A
**Anti-eIF3h**	Polyclonal / Rabbit	Bethyl / A301-754A
**Anti-eIF3i**	Monoclonal / Mouse	BioLegend / 646702
**Anti-eIF3k**	Polyclonal / Rabbit	Novus Biological / NB100-93304
**Anti-eIF3l**	Polyclonal / Rabbit	GeneTex / GTX120119
**Anti-GAPDH**	Monoclonal / Mouse	GeneTex / GTX627408
**Anti-H1**	Polyclonal / Rabbit	Neobiotech / NB-22-3513
**Anti-H2A**	Polyclonal / Rabbit	Neobiotech / NB-22_3516
**Anti-H2B**	Polyclonal / Rabbit	Neobiotech / NB-22-3519
**Anti-H3**	Polyclonal / Rabbit	Neobiotech / NB-22-3524
**Anti-H4**	Polyclonal / Rabbit	Neobiotech / NB-22-3534
**Anti-GST- HRP**	Polyclonal / Rabbit	GE Healthcare / RPN1236V
**Anti-eIF4E**	Monoclonal / Mouse	Santa Cruz / sc-271480
**Anti-SLBP**	Polyclonal / Rabbit	Bethyl / A303-968A
**HRP anti-Rabbit IgG**	Polyclonal / Goat	Bethyl / A120-101P
**HRP anti- Mouse IgG**	Polyclonal / Goat	Bio-Rad / 170-6516

Table S3: Oligonucleotides used for qRT-PCR experiments

Target	qRT-PCR oligonucleotides
H1	Fw: CGCTACCCCGAAGAAAAGCA<br>Rev: ACCTTTTTCGCACTCTTGGC
H2A	Fw: CGCAACGACGAGGAGCTAA<br>Rev: AAGAGTCTCGTTTTACTTGCCC
H2B	Fw: GTGACCAAGGCGCAGAAGAAGGAC<br>Rev: TTTAGAGGAGATGCCGGTGTCGGG
H3	Fw: CGTGAAAAAGCCTCACCGTT<br>Rev: TCCGAATCAGCAACTCGGTC
H4	Fw: AGGTGCTGCGGGACAATATC<br>Rev: GCCGAAACCATAAAGGGTGC
C-JUN	Fw: TGACTGCAAAGATGGAAACG<br>Rev: CAGGGTCATGCTCTGTTTCA
LDHA	Fw: TGGCAGCCTTTTCCTTAGAACA<br>Rev: ACGGCTTTCTCCCTCTTGCTGA
GAPDH	Fw: CTTTGGTATCGTGGAAGGACT<br>Rev: CCAGTGAGCTTCCCTTTTCAG
HPRT	Fw : TGACACTGGCAAAACAATGCA<br>Rev : GGTCCTTTTCACCAGCAAGCT
PGK1	Fw: GAAGCGGGTCGTTATGAGAGTC<br>Rev: AGGACTACCGACTTGGCTCCAT
ACTB	Fw: CCTTCTACAATGAGCTGCGT<br>Rev: CTCCTTAATGTCACGCACGAT
snRNA U2	Fw: TTCTCGGCCTTTTGGCTAAG<br>Rev: CTCCCTGCTCCAAAAATCCA
eIF3c	Fw: ACCAAGAGAGTTGTCCGCAGTG<br>Rev: TCATGGCATTACGGATGGTCC
eIF3d	Fw: CTGGAGGAGGGCAAATACCT<br>Rev: CTCGGTGGAAGGACAAACTC
eIF3e	Fw: TTCTTCAATCACCCCAAAGG<br>Rev: TAGAACCTGCCGACGTTTTC
eIF3g	Fw: TCTCGTTTTCGCCCGCTTC<br>Rev: GTCTCCAGTAGGCATCGCAA

Table S4: List of siRNA library (ON-TARGET plus SMART pools, Dharmacon)

**Target**	**GENE ID**	**Gene accession number**	**Sequence** (5’ -> 3’)
EIF3C	8663	NM_001199142	GCGGAGUGCCGUUGGUUAA<br>GGAGAGGGCGUCAUUGUCA<br>CAACUGCUGGUUCAGAUUG<br>GCAAGUACAUCUACGCCAA
EIF3D	8664	NM_003753	GUUCAAGCCUAAUGAGUUU<br>CAAGAUAAGAGGUACACAA<br>AGACAAAGAUCGUCGGAAC<br>CGAAUGAGAUUUGCCCAGA
EIF3E	3646	NM_001568	UGGCUUGUCUUGAGGAUUU<br>GGAUCGGCAUCUAGUCUUU<br>GGGUAACAAUGCAGUCUCA<br>AAAGGUCGCGAUAAUAUUA
EIF3G	8666	NM_003755	GGUCAUCAACGGAAACAUA<br>GUCCGCACCUUCAGGAUUG<br>GGACAAGACCACUGGCCAA<br>GACGAUGUCUCUAUGACGU
EIF4E	1977	NM_001968	GACGAUGGCUAAUUACAUU<br>CAUAUCCAGUUGUCUAGUA<br>GUGAUAAGAUAGCAAUAUG<br>GAGACGAAGUGACCUCGAU
SLBP	7884	NM_001306075	GAAAUGAGGACCAGAGUUA<br>GAAGUAUAGUCGACGUUCA<br>CGGCUGACUUUGAGACAGA<br>GACAGAAGCAGAUCAACUA
sicontrol	-	-	UGGUUUACAUGUCGACUAA<br>UGGUUUACAUGUUGUGUGA<br>UGGUUUACAUGUUUUCUGA<br>UGGUUUACAUGUUUUCCUA
